# Calcium signaling positively regulates cellulase translation and secretion in a Clr-2-overexpressing, catabolically derepressed strain of *Penicillium funiculosum*

**DOI:** 10.1186/s13068-023-02448-3

**Published:** 2024-02-09

**Authors:** Anmoldeep Randhawa, Olusola A. Ogunyewo, Kamran Jawed, Syed Shams Yazdani

**Affiliations:** 1 Microbial Engineering Group, International Centre for Genetic Engineering and Biotechnology, New Delhi, 110067 India; 2 DBT-ICGEB Centre for Advanced Bioenergy Research, International Centre for Genetic Engineering and Biotechnology, New Delhi, 110067 India; 3 Present Address: AMITY University, Mohali, Punjab 140306 India

**Keywords:** Cellulase, Clr-2, Ssp1 CaMKK, Calcium, *Penicillium funiculosum*

## Abstract

**Background:**

Low-cost cellulase production is vital to sustainable second-generation biorefineries. The catabolically derepressed strain of *Penicillium funiculosum* NCIM1228 (*Pf*Mig1^88^ or ∆Mig1) secretes a superior set of cellulolytic enzymes, that are most suitable for 2G biorefineries. At a 3% (w/w) load, the ∆Mig1 secretome can release > 80% of fermentable sugars from lignocellulose at a 15% (w/v) biomass load, irrespective of the type of biomass and pretreatment. The robustness of the secretome can be further increased by improving the cellulase production capacity of the fungal strain.

**Results:**

We began by identifying the transcription factor responsible for cellulase production in NCIM1228. An advanced RNA-seq screen identified three genes, *clr-2*, *ctf1a* and *ctf1b*; the genes were cloned under their native promoters and transformed into NCIM1228. Of the three, *clr-2* overexpression led to twofold higher cellulase production than the parent strain and was thus identified as the transcriptional activator of cellulase in NCIM1228. Next, we overexpressed *clr-2* in ∆Mig1 and expected an exponential increase in cellulolytic attributes accredited to the reinforced activation mechanisms, conjoint with diminished negative regulation. Although *clr-2* overexpression increased the transcript levels of cellulase genes in ∆Mig1, there was no increase in cellulase yield. Even a further increase in the transcript levels of *clr-2* via a stronger promoter was ineffective. However, when the CaCO_3_ concentration was increased to 5 g/l in the growth medium, we achieved a 1.5-fold higher activity of 6.4 FPU/ml in the ∆Mig1 strain with *clr-2* overexpression. Enthused by the calcium effect, a transcriptomic screen for genes encoding Ca^2+^-activated kinase identified *ssp1*, whose overexpression could further increase cellulase yield to ~ 7.5 FPU/ml. Investigation of the mechanism revealed that calcium signaling exclusively enhances the translation and secretion of cellulase in *Penicillium funiculosum*.

**Conclusions:**

Our study identifies for the first time that cellulose activates two discrete signaling events to govern cellulase transcription and posttranscriptional processes (translation, processing and secretion) in *P. funiculosum* NCIM1228. Whereas Clr-2, the transcriptional activator of cellulase, governs transcription, calcium signaling specifically activates cellulase translation and secretion.

**Supplementary Information:**

The online version contains supplementary material available at 10.1186/s13068-023-02448-3.

## Background

The bioconversion efficacy of lignocellulosic biomass and the cost associated with it are major challenges in a 2G-biorefinery setup [1]. The cost-effective production of superior lignocellulolytic enzymes is vital to sustainable bioconversion systems [2]. Owing to their ability to secrete biomass hydrolyzing enzymes, saprophytic fungi are ideal for the commercial production of cellulase. Our earlier bioprospecting efforts for a superior cellulolytic secretome identified *Penicillium funiculosum* NCIM1228. It secretes an exceptional lignocellulolytic enzyme assortment in cellulosic growth medium, which is most suitable for 2G biorefineries [3–5]. GH7 cellobiohydrolase (Cbh1) is the crucial enzyme for cellulose breakdown in filamentous fungi. *P. funiculosum* Cbh1 exhibited an 18-fold higher turnover rate, sixfold higher catalytic efficiency, and 26-fold higher enzyme-inhibitor complex equilibrium dissociation constant (K_i_) than Cbh1 of *T. reesei* [6]. Also known for higher β-glucosidase levels, the secretome of *P. funiculosum* is a sustainable substitute for *T. reesei*-based secretomes for wide-ranging industrial applications [4, 5]. We endorsed a targeted perspective to increase lignocellulase productivity of *P. funiculosum* NCIM1228 and constructed a customized molecular toolbox to carry out genetic engineering of the fungus [7].

The expression of lignocellulolytic enzymes is repressed by glucose and other fermentable sugars in filamentous fungi and is stringently activated in a substrate-specific manner during carbon stress [8–10]. Recent studies have identified both positive and negative regulatory circuits governing lignocellulase production at the transcriptional level [9–12]. A global regulator of secondary carbon metabolism, Mig1/Cre-1/CreA, also called catabolite repressor, responds to intracellular glucose concentrations and accordingly modulates the transcriptional dynamics of lignocellulolytic genes [13–17]. In our first attempt, the fungus was genetically engineered to replace the functional allele of the catabolite repressor *Pfmig1* with a *mig1* deletion cassette [18]. The resultant strain, ∆Mig1 (also called *Pf*Mig1^88^), exhibited catabolite derepression on most of the enzymes of alternate carbon utilization and yielded ~ 4 FPU/ml with more than a higher lignocellulase production [19]. We also overexpressed two of the key lignocellulolytic enzymes, *Pf*Cbh1 and *Pf*Aa9, in ∆Mig1 to further increase the saccharification abilities of its secretome [20].

The induction of cellulose hydrolyzing enzymes is governed at the transcriptional level by positive regulators [11, 21, 22]. These transcriptional activators of cellulase vary among filamentous fungi; Xyr1 is the key transcriptional activator in *T. reesei *[23], and Clr-1 and Clr-2 are a set of conserved transcriptional activators in *Neurospora crassa *[11]. In *Aspergillus niger*, XlnR, ClrB, and to a lesser extent, ClrA regulate cellulase expression upon induction [2, 24]. Similar to archetypical Gal4 transcription factors in yeast, Clr-1, Clr-2, and Xyr1 are members of the Zn(II)_2_Cys_6_ family of transcription factors with Zn–Cys binuclear cluster-type DNA-binding domains and activators of genes of secondary carbon sources. In addition, homologs of Ace3, Bgl-R, Crz1, Vib1, etc., have also been identified as transcriptional activators of lignocellulase in filamentous fungi [11, 12, 25–27]. Furthermore, lignocellulase production is also influenced by environmental factors such as Ca^2+^, light, and pH under carbon stress [25, 28, 29].

In the present study, we aimed to reinforce the positive regulatory networks to achieve higher production of lignocellulase. Since the key transcriptional activator of cellulase varies among different fungi, we began by identifying the transcription factor responsible for cellulase production in *P. funiculosum* NCIM1228. Advanced transcriptomics identified Clr-2 as the key transcriptional activator of cellulase in *P. funiculosum* in response to induction by the polymeric substrate cellulose. Functional studies of *clr-2*-overexpressing mutants revealed the limited role of Clr-2 beyond cellulase transcription. We achieved a synergistic effect of *clr-2* overexpression and *mig1* deletion by increasing the Ca^2+^ concentration in the growth medium. We next studied the effect of Ca^2+^-signaling on cellulase transcription, translation, and secretion and found that the presence of both cellulose and calcium was necessary for cellulase translation and secretion. A transcriptomic screen for Ca^2+^-activated kinase identified the gene encoding calcium/calmodulin-dependent kinase kinase (CaMKK) Ssp1, whose over-expression further escalated lignocellulase production by 40%. Calcineurin-dependent calcium signaling has been shown to upregulate the gene expression of the transcriptional activator Xyr1 via the calcineurin-responsive zinc finger transcription factor Crz1 [25, 30]; however, this is the first time that the synergistic effect of *clr-2* overexpression and *mig1* deletion was achieved by re-enforcing Ca^2+^ signaling events. We further strengthened the positive effects of calcium signaling by overexpressing *ssp1*. This resulted in an engineered strain of *P. funiculosum* NCIM1228 with an escalated transcriptional response to cellulose along with enhanced protein translation and secretion capabilities, resulting in > sevenfold increase in lignocellulase production compared with the parent strain *P. funiculosum* NCIM1228.

## Results

### Screen for transcriptional activators of lignocellulase in *P. funiculosum* NCIM1228

**Fig. 1:**
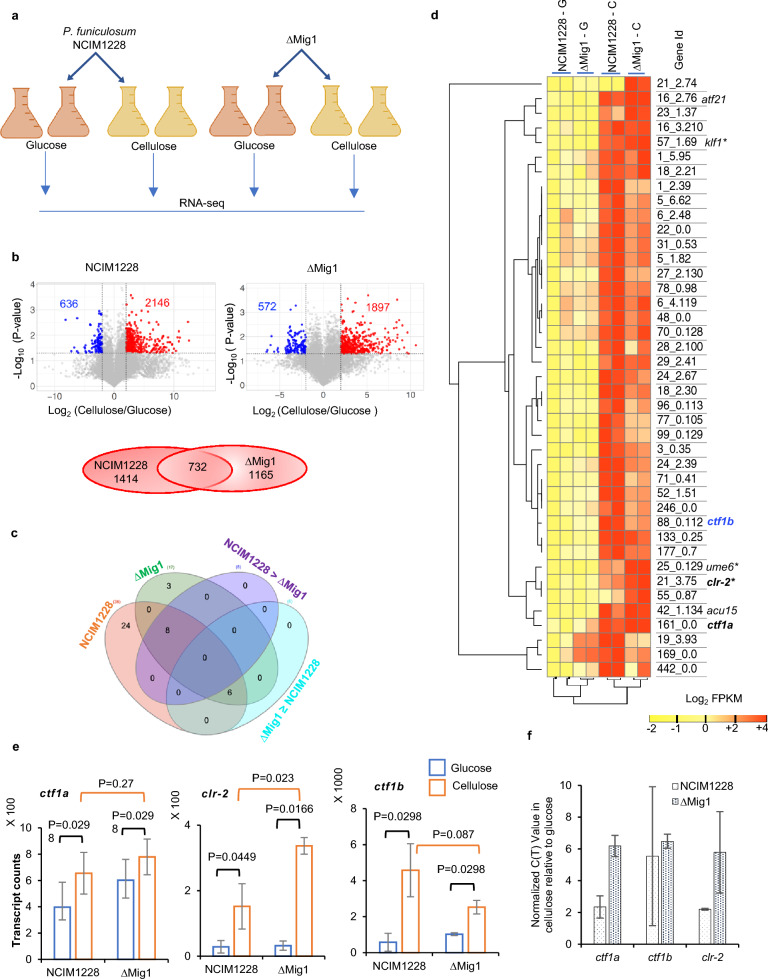
Cellulose-induced transcription factors in *P. funiculosum* NCIM1228 and ∆Mig1 identified by RNA-seq. **a** RNA-seq experimental design to conduct transcriptomic analysis of NCIM1228 and ∆Mig1 grown in Mandel's medium with 4% glucose (MM glucose) and 4% cellulase (MM cellulose) for 72 h. **b** Volcano plot of differentially expressed genes in cellulose with respect to glucose in NCIM1228 and ∆Mig1. Venn diagram showing the commonly upregulated genes between NCIM1228 and ∆Mig1. **c** Venn diagram of common and exclusive transcription factors (TFs) upregulated in NCIM1228 and ∆Mig1; 8 of the common TFs showed a higher log2-fold change in NCIM1228 than in ∆Mig1, whereas 6 of them showed equivalent or higher expression in ∆Mig1. **d** Hierarchical (left) clustering of normalized FPKM abundance (log_2_) of 41 TFs in NCIM1228 and ∆Mig1 grown on glucose and cellulose. Three transcription factors related to biomass degradation were identified: Clr-2 (TF for cellulase) and Ctf1a and Ctf1b (TFs for cutinase). **e** Transcript counts of *ctf1a*, *clr-2*, and *ctf1b* in NCIM1228 and ∆Mig1 grown in glucose and cellulose. Statistical significance was determined by a one-tailed, unequal variance t test. **f** Quantitative RT‒PCR of selected TFs in NCIM1228 and ∆Mig1 grown in MM glucose and MM cellulose for 72 h. The C(T) value in cellulose was normalized to that in glucose

We aimed to identify the key transcription factors (TFs) involved in the regulation of cellulase gene expression in *P. funiculosum*. We analyzed the global transcriptome of log-phase cultures of NCIM1228 and ∆Mig1 grown in glucose and cellulose by RNA-seq (Fig. 1a, b). Comparative analysis of cellulose/glucose detected upregulation of 38 TFs in NCIM1228 and 17 TFs in ∆Mig1 in cellulose, while 14 of these TFs were detected in both (Fig. 1c, d). We argued that TFs responsible for cellulase transcriptional activation must be induced in both NCIM1228 and ∆Mig1. Therefore, 24 TFs exclusive to NCIM1228 and 3 TFs (21_2.74, 23_1.37, 55_0.87) exclusive to ∆Mig1 were ignored (Fig. 1c, d). Further, the putative TFs involved in transcriptional activation of cellulase should exhibit higher or equivalent transcript levels in catabolically derepressed ∆Mig1, and we found six common TFs aligning with our hypothesis (i.e., ∆Mig1 ≥ NCIM1228 in Fig. 1c). Among these, 3 TFs had equal transcript levels in both NCIM1228 and ∆Mig1 and were identified as homologs of *ctf1a* (TF for cutinase) [31], *acu15* (TF for acetate utilization) [32], and *atf21* (TF for spore maturation) [33, 34]. The other three common TFs demonstrated higher levels in ∆Mig1 and were found to be homologs of *clr-2* (TF for cellulase) [10, 11], *ume6* (TF for early meiotic genes) [35], and *klf1* (TF for G0 phase longevity) [36] (Fig. 1d). Of the six shortlisted TFs, *clr-2* and *ctf1a* seemed relevant to cellulase induction and, therefore, were chosen for further analysis (Fig. 1e). Additionally, we also shortlisted 88_0.112 (*ctf1b*) for having 33% homology to the cutinase regulator of *Fusarium solani* [37] (Fig. 1e). We reconfirmed the transcript levels of the three shortlisted TFs in glucose and cellulose by real-time PCR and found them in sync with the transcriptomic results (Fig. 1f).

### Overexpression of *clr-2*, *ctf1a*, and *ctf1b* in *P. funiculosum* NCIM1228

**Fig. 2:**
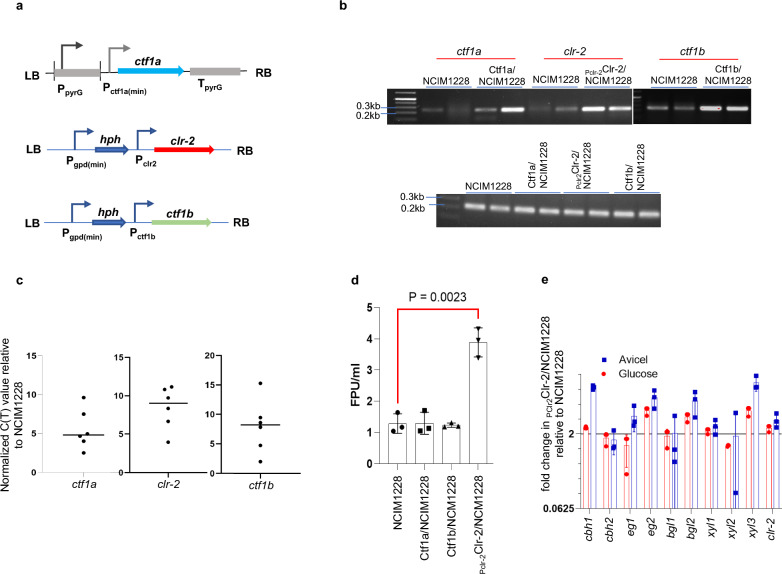
Clr-2 is the transcriptional activator of lignocellulase in *P. funiculosum*. **a** Schematic representation of expression cassettes of *ctf1a*, *clr-2*, and *ctf1b*. **b** Transcript levels of *ctf1a*, *clr-2*, and *ctf1b* reflected in their respective overexpressed strains by semiquantitative PCR. The lower panel shows the technical replicates of tubulin expression in all the strains. **c** Transcript levels of *ctf1a* (left), *ctf1b* (middle), and *clr-2* (right) in their respective overexpressed strains measured by real-time PCR in the corresponding transformants. **d** Total cellulase activity of secretomes measured by filter paper unit (FPU) assay. **e** Transcript levels of cellulases in P_clr2_Clr-2/NCIM1228 relative to NCIM1228 when grown in MM glucose and MM cellulose for 72 h

Genes encoding Clr-2, Ctf1a, and Ctf1b were overexpressed individually in NCIM1228 cells under their native promoters. The expression cassettes were constructed by PCR and cloned and inserted into the pBIF expression vector [7] (Fig. 2a–c, Additional file 2: Figures S1and S2) and were eventually transformed into NCIM1228. The transformants were verified for having an additional copy of the respective gene by PCR (Additional file 2: Figures S1 and S2). To check the consistent pattern of positive transformants, at least three positive transformants were checked for the cellulase production in the RCM medium. Out of the transformants showing similar phenotype, the best performing transformant was selected for further analysis (Additional file 2: Figure S3). The increased levels of respective transcripts were confirmed by real-time PCR in best-performing mutants (Fig. 2c). For comparative studies, the best-performing *clr-2*, *ctf1a*, and *ctf1b* over-expression mutants were grown in cellulosic medium, and the secretomes obtained were examined for total cellulase activity by filter paper unit (FPU) assay (Fig. 2d). Of the three, P_clr2_Clr-2/NCIM1228 exhibited twofold higher FPU levels than NCIM1228; even transcript levels of key cellulase genes, namely, cellobiohydrolase1 (*cbh1*), endoglucanase (*eg2*), β-glucosidase (*bgl2*) and xylanase (*xyl3*), were upregulated in both glucose and cellulose (Fig. 2e). Overexpression of *ctf1a* and *ctf1b* did not have any effect on cellulase or the biomass hydrolyzing capacity of the NCIM1228 secretome; however, cutinase activity decreased and the two transcription factors behaved as repressors of cutinase under cellulosic conditions (Additional file 2: Figure S4).

### Effect of Clr-2 overexpression in ∆Mig1

**Fig. 3:**
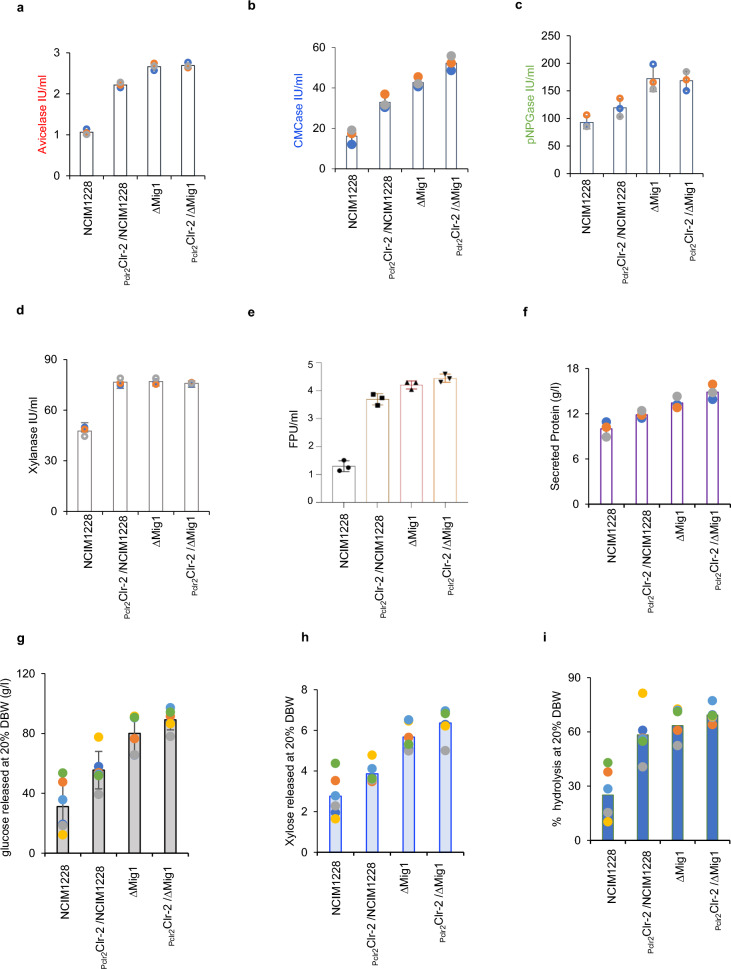
Effect of *clr-2* overexpression in ∆Mig1. *p*P_clr2_Clr-2 was transformed into ∆Mig1, and the secretome of the resultant strain, P_clr2_Clr-2/∆Mig1, was evaluated for **a** exocellulase (Avicelase), **b** endocellulase (CMCase), **c** β-glucosidase (pNPGase), and **d** xylanase along with the secretomes of NCIM1228, P_clr-2_Clr-2/NCIM1228, and ∆Mig1. **e** Total cellulase activity of the P_clr2_Clr-2/∆Mig1 secretome compared with parent strains by FPU assay. **f** Total protein present in the secretomes was measured by the BCA method. The biomass hydrolyzing capacity of secretomes was determined by incubating 20% nitric acid-pretreated rice straw biomass (by dry biomass weight (DBW)) with secretomes at a 30 mg/ml concentration for 72 h. The supernatant collected was used to determine glucose **g** and xylose **h** release by HPLC. Percentage hydrolysis **i** was calculated by dividing the total sugar released during hydrolysis by the total sugar content present in the dry biomass

∆Mig1 is a catabolically derepressed strain of *P. funiculosum* with weakened negative regulation. ∆Mig1 secretes a greater amount of lignocellulase than the parent strain in the presence of cellulose. With the intent of further increasing lignocellulase production, we overexpressed *clr-2* under its native promoter in the ∆Mig1 strain. We expected an exponential increase in cellulolytic enzyme content in the secretome, credited to the reinforced activation mechanisms combined with weakened negative regulation [38, 39]. The P_clr2_Clr-2/∆Mig1 secretome was tested for individual classes of enzymes, namely, exocellulase (Avicelase), endocellulase (CMCase), β-glucosidase (pNPGase), and xylanase (Fig. 3a–d). Exocellulase, β-glucosidase and xylanase activity remained the same in the secretomes of ∆Mig1 and P_clr2_Clr-2/∆Mig1; there was a marginal increase in endocellulase activity in P_clr2_Clr-2/∆Mig1 (Fig. 3a–d). We also examined the total cellulase activity by FPU assay and found no significant difference between the activities of P_clr2_Clr-2/NCIM1228, ∆Mig1, and P_clr2_Clr-2/∆Mig1 (Fig. 3e). The double mutant showed marginal supremacy over the parent strains. Similar results were obtained when nitric acid-pretreated rice straw was hydrolyzed by the secretomes of parent and mutant strains (Fig. 3f–h); the release of sugar and percentage hydrolysis by secretomes were found to be in the order NCIM1228 < P_clr2_Clr-2/NCIM1228 < ∆Mig1 ≤ P_clr2_Clr-2/∆Mig1. To conclude, we did not find a statistically significant difference in the secretome performance of ∆Mig1 and P_Clr2_Clr-2/∆Mig1 (Fig. 3).

### Optimized *clr-2* overexpression in ∆Mig1

**Fig. 4:**
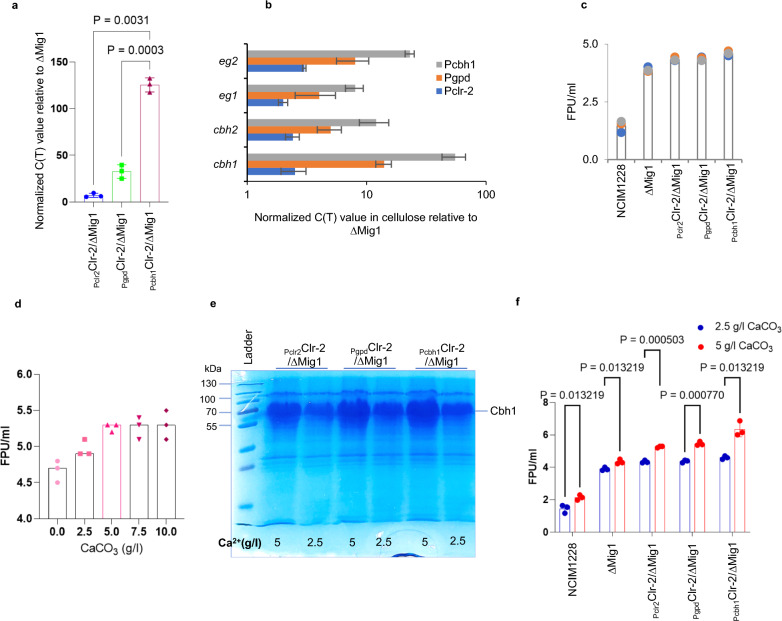
*clr-2* overexpression leads to increased cellulase when complemented with increased Ca^2+^ in the growth medium. RT‒PCR showing transcript levels of **a** *clr-2* and **b** cellulase genes when expressed under native, *gpd* (constitutive), and *cbh1* (inducible) promoters. **c** Total cellulase activity of secretomes obtained when *clr-2*-overexpressing strains were grown in RCM medium. **d** Total cellulase activity of secretomes obtained when P_clr2_Clr-2/∆Mig1 was grown in RCM medium with varying levels of CaCO_3_. Secretome profile on 12% SDS‒PAGE **e** and total cellulase activity **f** by FPU assay of *clr-2* mutants grown in RCM medium with 2.5 g/L and 5.0 g/L CaCO_3_. All experiments were repeated three times; statistical significance was determined by a one-tailed, unequal variance t-test

Dwelling further into the transcriptomic data made us realize extremely low levels of *clr-2* transcript compared with housekeeping genes. Therefore, we overexpressed *clr-2* under the *Pfcbh1* inducible promoter (the top-ranked gene expressed in cellulose) and a strong constitutive *gpd* promoter (Additional file 2: Figure S2). Quantification by real-time PCR found *clr-2* transcript levels in the order P_cbh1_ > P_gpd_ > P_clr-2_ (Fig. 4a); a similar pattern was observed in the transcripts for downstream Clr-2-regulated cellulases (Fig. 4b). However, differential expression of the *clr-2* transcript and cellulases had minimal effects on the secretion levels of the cellulases (Fig. 4c). Bioinformatic analysis of the Clr-2 protein predicted a Gal4 DNA binding domain (35–80 aa), nuclear localization signal [40] (45–65 aa) and fungal TF-specific domain (355–436 aa). A recent study on Clr-2 suggested the removal of the middle regulatory region of Clr-2 (248–646 aa) to increase cellulase production [41]. In our case, however, a similar strategy resulted in reduced cellulase levels in the ∆Mig1 strain; perhaps the removal of the regulatory region from Clr-2 made it nonfunctional (Additional file 2: Figure S5).

### Increased Ca^2+^ levels in the growth medium relieve the posttranscriptional bottleneck in cellulase production

Studies on *Trichoderma reesei* indicated the role of Ca^2+^ signaling in hyphal growth and cellulase induction [28, 30, 42]. In our previous reports, we used 50 mg/l CaCl_2_ or 0.5 g/l CaCO_3_ in cellulase-inducing RCM medium [18, 19]. We next reasoned that increasing Ca^2+^ levels would impact cellulase production[28, 43]. Therefore, we cultured P_clr2_Clr-2/∆Mig1 in RCM medium containing 50 mg/l CaCl_2_ with varying levels of CaCO_3_ from 0 to 10 g/l and achieved a maximum activity of 5.3 FPU/ml at 5 g/l (Fig. 4d). Next, we cultured all three *clr-2* overexpression mutants in RCM medium containing 5 g/l CaCO_3_ and obtained heightened cellulase levels in the order P_clr-2_ < P_gpd_ < P_cbh1._ The P_cbh1_Clr-2/∆Mig1 secretome had a maximum cellulase activity of 6.3 FPU/ml (Fig. 4e, f). Increasing the calcium concentration complemented the increase in *clr-2* transcripts and delivered higher cellulase production in the same order.

### Ca^2+^ regulates cellulase production at the protein level

The addition of CaCO_3_ to the growth medium also leads to a pH buffering effect during fungal growth. To rule out whether the increase in cellulase is attributed to a more neutral pH of the medium by the addition of CaCO_3_ and not necessarily to an increase in calcium ions, we performed our next set of experiments solely in the presence of CaCl_2_.

**Fig. 5:**
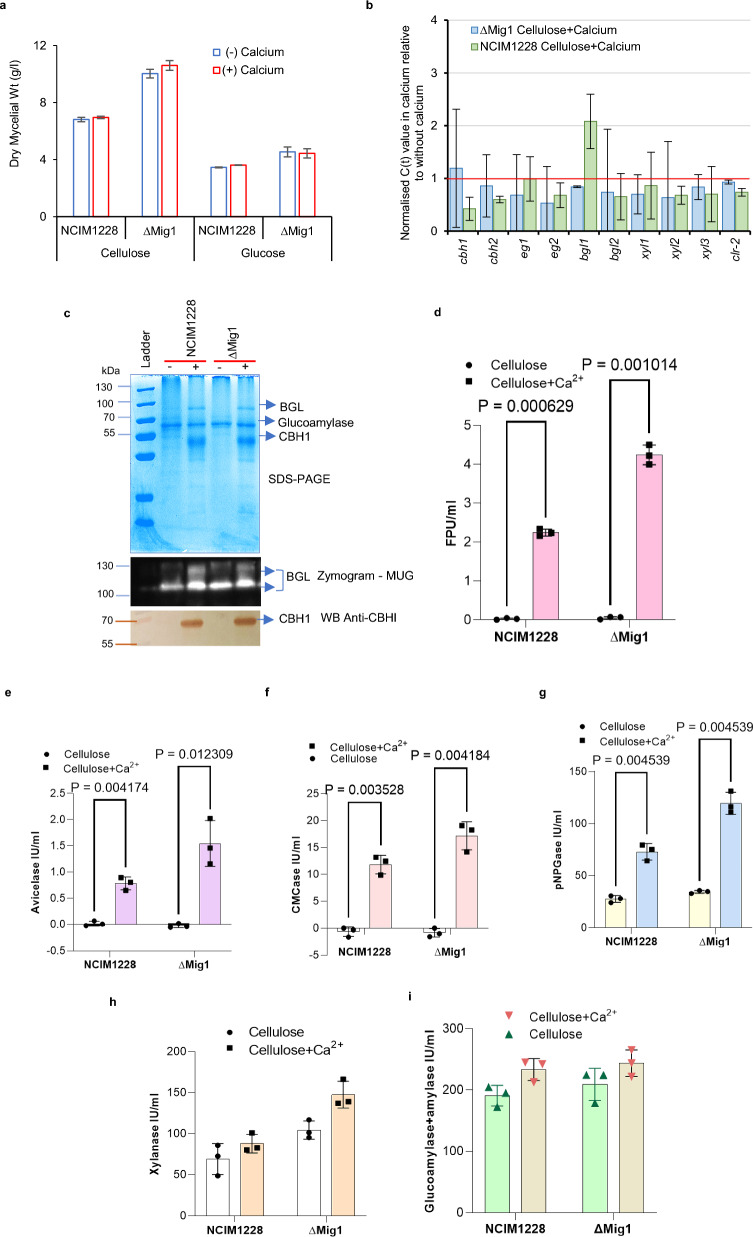
Posttranscriptional events leading to cellulase production are Ca^2+^ dependent. **a** Dry mycelial weight of 24-h cultures of NCIM1228 and ∆Mig1 in Mandel's medium containing 4% glucose and 4% cellulose with and without 50 mg/l CaCl_2_. **b** Impact of calcium on transcript levels of important cellulases in NCIM1228 and ∆Mig1 grown in glucose (30 h) and cellulose (60 h) by RT‒PCR. **c** SDS‒PAGE, in-gel MUG β-glucosidase zymogram, and Western blot with anti-Cbh1 of NCIM1228 and ∆Mig1 secretome showing the effect of calcium on total secretome profile, β-glucosidase and Cbh1 levels, respectively. NCIM1228 and ∆Mig1 secretomes were also examined for **d** total cellulase activity by filter paper unit (FPU) assay, **e** exocellulase (cellulase), **f** endocellulase (CMCase), **g** β-glucosidase (pNPGase), **h** xylanases, and **i** total amylase activity. All experiments were repeated three times; statistical significance was determined by a one-tailed, unequal variance t test

To understand the role of Ca^2+^ in cellulase production, we first examined whether the lack of Ca^2+^ affects hyphal growth in glucose and cellulose. The effect was negligible in both NCIM1228 and ∆Mig1 (Fig. 5a). Notably, even transcriptional induction of *clr-2* and lignocellulase remained unaffected in the absence of Ca^2+^ in cellulosic medium (Fig. 5b). However, both NCIM1228 and ∆Mig1 grown without calcium secreted negligible levels of key cellulases (Fig. 5c). An in-gel fluorescent (MUG) assay and Western blotting with anti-Cbh1 antibody confirmed reduced β-glucosidase and Cbh1 levels, respectively (Fig. 5c). FPU activity fell to near zero levels due to the absence of exocellulase and endocellulase in the secretome (Fig. 5d). Quantitative evaluation of the secretomes for four major cellulolytic enzyme classes (exocellulase (Avicelase), endocellulase (CMCase), β-glucosidase (pNPGase), and xylanase) revealed reduced enzyme activities in the order exocellulase > endocellulase > β-glucosidase > xylanases (Fig. 5e–h). Notably, glucoamylase secretion remained unaffected (Fig. 5c, i). Therefore, calcium specifically regulated the post-transcriptional events (translation and secretion) of cellulolytic enzymes in *P. funiculosum* NCIM1228. This was surprising, as this phenomenon has not been reported until now for any of the cellulase-producing filamentous fungi.

### Ssp1 is a carbon-derepressing CaMKK

**Fig. 6:**
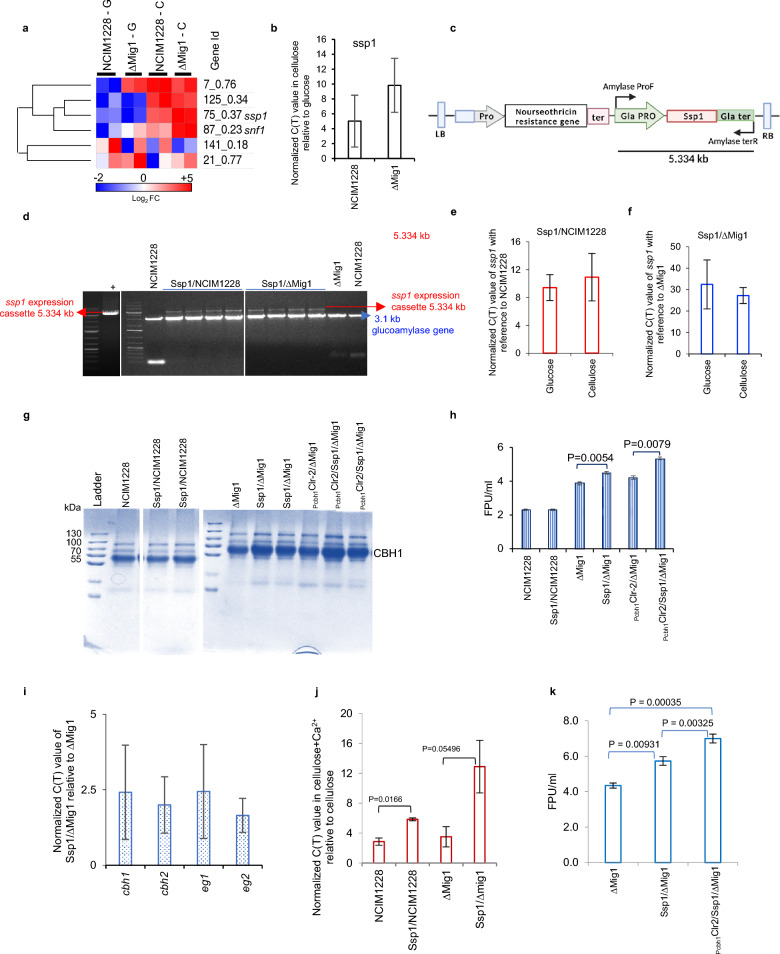
Ssp1 is a carbon-derepressing Ca^2+^-activated kinase. **a** Heatmap of differentially regulated Ca^2+^-activated kinases identified in NCIM1228 and ∆Mig1. **b** An increase in transcript levels of *ssp1* in NCIM1228 and ∆Mig1 was determined by RT‒PCR in cellulose relative to glucose. **c** Schematic of the *ssp1* expression cassette. **d** Confirmation PCR of *ssp1* expression cassette integration. Transcript levels of *ssp1* when Ssp1 was overexpressed in **e** NCIM1228 and **f** ∆Mig1. **g** SDS‒PAGE profile and **h** total cellulase activity present in the secretomes of parent and engineered strains grown in cellulosic RCM medium. **i** Transcript levels of major cellulases in Ssp1/∆Mig1 compared to ∆Mig1 in MM medium containing cellulose. **j** Transcript levels of *ssp1* in cellulose + Ca.^2+^ relative to cellulose. **k** Total cellulase activity of Ssp1/∆Mig1 and P_cbh1_Clr-2/Ssp1/∆Mig1 determined by FPU assay when the strains were grown in RCM medium with 5 g/l CaCO_3_

We next hypothesized that calcium might be influenced through a heightened signaling response to cellulose. We next mined the transcriptomic data for any genes of Ca^2+^-activated kinase exclusively upregulated in cellulose. We found the gene ID 75_0.37 encoding the most significant CaMKK with ~ fivefold higher transcript levels in cellulose than glucose in ∆Mig1 in RNA-seq data (Fig. 6a). Gene sequence analysis of 75_0.37 showed its homology to *Schizosaccharomyces pombe* Ssp1 protein with 60% identity. Ssp1 is known to regulate the CaMKK-AMPK signaling cascade by phosphorylating Ssp2 AMPK (AMP activated protein kinase) in *S. pombe* [44, 45]. Ssp1 was also found to be involved in the negative regulation of TORC1 signaling and the cellular response to nitrogen starvation [46, 47]. We hypothesized that if Ssp1 is activated by Ca^2+^ and is involved in cellulase translation and secretion under inducing conditions, its overexpression should increase the cellulase yield of NCIM1228.

Real-time PCR of *ssp1* in NCIM1228 and ∆Mig1 detected extremely low transcript levels in glucose compared to tubulin, which increased by five to ninefold in cellulose (Fig. 6b). To increase the transcript levels of *ssp1* in both glucose and cellulose, we screened the transcriptomic data for a highly expressed promoter in glucose. Glucoamylase promoter was the most expressed promoter under repressing conditions in both NCIM1228 and ∆Mig1 (Additional file 2: Figure S6). We cloned the 4317 bp *ssp1* gene of *P. funiculosum*, encoding a 133 kDa protein, under the native glucoamylase promoter in a binary vector and transformed it into NCIM1228 and ∆Mig1 (Fig. 6c). Transformants were confirmed to have an additional copy of *ssp1* by PCR (Fig. 6d), and the total cellulase activity of at least three confirmed transformants was conducted to achieve a consistent phenotype associated with the over-expression mutants (Additional file 2: Figure S6). The increased transcript levels of *ssp1* in glucose and cellulose were confirmed by real-time PCR (Fig. 6e, f). The SDS‒PAGE profile and FPU assay of secretomes reflected no change in cellulase production by Ssp1/NCIM1228 compared to NCIM1228, but Ssp1/∆Mig1 secretomes exhibited 40% higher FPU levels than ∆Mig1 (Fig. 6g, h). We did not find any noticeable change between the cellulase transcript levels of Ssp1/∆Mig1 and ∆Mig1, suggesting that the reason for increased activity might be at the protein level (Fig. 6i). We also examined the effect of Ca^2+^ on *ssp1* transcript levels and cellulase production by SSP1/∆Mig1. We indeed found increased *ssp1* transcripts in cellulose + Ca^2+^ relative to cellulose (Fig. 6j); moreover, total cellulase activity was increased to ~ 6 FPU/ml in Ssp1/∆Mig1 grown in cellulosic RCM medium with 5 g/l CaCO_3_ (Fig. 6k). We also overexpressed *ssp1* in P_cbh1_Clr-2/∆Mig1 and achieved ~ 7.3 FPU/ml in its secretome obtained from RCM medium with 5 g/l CaCO_3_ (Fig. 6k). This confirms the regulatory role of Ca^2+^ signaling in cellulase translation and secretion, and reinforcing the signaling pathway resulted in increased extracellular cellulase production which is sevenfold higher than the parent strain *P. funiculosum* NCIM1228.

## Discussion

The response of filamentous fungi toward recalcitrant carbohydrates is manifested by the release of cellulase into the surroundings. Various studies in filamentous fungi have identified transcriptional activators of cellulase; however, little is known about the downstream events beyond transcription. The present study was conducted with the aim of identifying the transcription factors and signaling networks governing the extracellular production of lignocellulolytic enzymes in *P. funiculosum* NCIM1228.

**Fig. 7:**
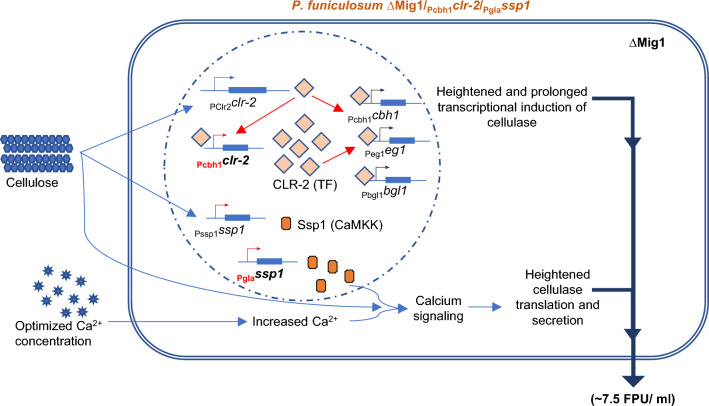
Enhanced induction of cellulase at transcriptional and translation and secretory levels. We achieved a > sevenfold increase in cellulase yield compared with the parent strain NCIM1228 by (i) strategic removal of catabolite repression (∆Mig1), ii *clr-2* overexpression through a positive feedback loop, iii increased Ca.^2+^ concentration in the induction medium, and iv *ssp1* overexpression. These changes weakened catabolite repression and enhanced transcriptional induction and the translation and secretion of cellulase in *P. funiculosum* NCIM1228

Filamentous fungi harbor the Zn(II)_2_Cys_6_ family of transcription factors, which are involved in the induction of genes of secondary carbon sources. Our advanced RNA-seq screen followed by overexpression studies identified Clr-2 as the transcriptional activator of cellulase in *P. funiculosum* NCIM1228. Clr-2 is a conserved TF involved in cellulase induction in filamentous fungi and was first identified in *N. crassa and Aspergillus nidulans* [11]. Our study elucidates that cellulose alone is sufficient to induce transcriptional activation of cellulase via Clr-2 in *P. funiculosum*; however, the twin signal of cellulose + Ca^2+^ is vital to cellulase translation and secretion (Fig. 7). Furthermore, increasing the transcript levels of *clr-2* is effective only when downstream Ca^2+^ signaling is also accelerated to achieve homeostasis between cellulase transcription and translation (Fig. 7). *clr-2* expression was most effective under the *cbh1* promoter, probably because of the positive feedback loop formed during cellulase-inducing conditions [48].

We next screened our transcriptomics data for Ca^2+^-activated kinase and identified a gene encoding for CaMKK Ssp1, which is upregulated during carbon stress. Since *ssp1* overexpression improved cellulase yield in the ∆Mig1 secretome but not in NCIM1228, degradation of Mig1 during carbon stress seemed essential to maintain *ssp1* in its activated state (Fig. 7). In *A. nidulans*, CreA (Mig1 homolog) is phosphorylated at a conserved S319 site in the absence of glucose and other fermentable sugars, leading to its degradation [13, 49]. Further, Mig1 homologs are also known to get phosphorylated by Snf1 AMPK during carbon stress, leading to its inactivation in yeast and higher eukaryotes [45–47]. Degradation of Mig1 marks the arrival of carbon stress, and fungi start responding to the presence of alternate, nonfermentable carbon sources such as cellulose [17, 49]. We believe that once cellulose is converted into glucose, Mig1 restores carbon repression leading to activation of protein kinase A. Protein Kinase A is known to inhibit CaMKK in higher eukaryotes [50, 51], and similar events in *P. funiculosum* might prevent Ca^2+^-signaling and stop the further production and release of cellulase into the surroundings. In ∆Mig1, carbon repression is defective, leading to prolonged activation of Ssp1 activated pathways and increased cellulase production.

Ssp1 regulates cell proliferation and the coordination of meiosis with spore formation in *S. pombe*; we believe that these processes are linked to cellulase production in filamentous fungi. First, because exocytic secretion solves the dual purpose of secreting cellulase to the exterior while propagating hyphal growth toward crystalline cellulose, and second, cellulose, being the most recalcitrant carbon source, must be the last available carbon source in the proximal environment and therefore might be a signal of the arrival of unfavorable conditions, inducing meiosis. Furthermore, Ssp1 is known to phosphorylate Ssp2 AMPK, which negatively regulates the mTORC1-mediated pathway during nutritional stress in *S. pombe* [45, 46, 52]. Additionally, AMPK regulates Mig1 as well as energy homeostasis during starvation conditions, and we believe that the same events might also occur in *P. funiculosum* during carbon repression [53, 54]. Studies on CaMKK homologs in higher eukaryotes indicate CaMKKs are the most upstream element of a CaM-kinase cascade and are autoinhibited [55–57]. The autoinhibition is relieved by binding of Ca^2+^/calmodulin and initiation of phosphorylation events of downstream CaMKI, CaMKII, and AMPK [55–57]. CaMKK phosphorylates calcium kinases CaMK1 and CaMK2 which activate Ca^2+^-signaling in the eukaryotes [55–57].

Studies on filamentous fungi such as *T. reesei* and *N. crassa* have shown that calcium signaling regulates cellulase transcription via the calcium responsive regulator Crz1. However, we did not find any effect of Ca^2+^ signaling on cellulase transcript levels in *P. funiculosum*. Our study reports a unique Ca^2+^-dependent mechanism governing the translation and secretion of cellulase in *P. funiculosum* NCIM1228. We observed segregated control of cellulase production in *P. funiculosum* at the transcriptional and posttranscriptional levels. Furthermore, reinforcing both regulatory pathways is essential to achieve high lignocellulase yield. To summarize, we found cellulase production and secretion to be highly unique and coordinated events in *P. funiculosum*. It is indeed intriguing how conserved pathways such as Ca^2+^ signaling adopt different functions and mechanisms of action during evolution.

## Conclusions

Our study elucidates that cellulose alone is sufficient to induce transcriptional activation of cellulase via Clr-2 in *P. funiculosum* during carbon stress; however, the twin signal of cellulose + Ca^2+^ is vital to cellulase translation and secretion. Furthermore, increasing the transcript levels of Clr-2 is effective only when downstream Ca^2+^ signaling is also accelerated to achieve homeostasis between cellulase transcription and translation. We also identified Ssp1, a CaMKK, whose overexpression increased cellulase levels in ∆Mig1 but not in NCIM1228. Integrating promoter engineering of genes of transcription factors with reinforcing protein translation and secretory machinery is a promising strategy to improve lignocellulase yield in filamentous fungi.

## Materials and methods

### Strains, plasmids, growth media and culture conditions

All strains, plasmids and primers used in this study are listed in the Additional file 2: Tables S1, S2, and S3, respectively. All fungal strains are derivatives of *P. funiculosum* NCIM1228. Gene overexpression in *P. funiculosum* was carried out as previously described [7].

The growth media used in the study were potato dextrose (PD) broth and agar (Himedia, Thane, India) for conidiospore germination, low malt extract peptone (LMP) agar [7] for transformations, Mandel’s medium (MM) for transcriptomics, RNA profiling by RT‒qPCR, and RCM/MM medium for cellulase production [18, 19].

Primary cultures were prepared by adding 10^6^ conidiospores to 50 ml of PD broth, and the cultures were allowed to grow at 28 °C for 30 h at 120 rpm. Secondary cultures are inoculated with 10% primary cultures. The growth profile on agar plates was observed by spotting 5 μl of 10^4^ conidiospores/ml suspension on SC agar with a 2% carbon source followed by 48 h of incubation at 28 °C. To determine the differential growth profile in liquid medium, Mandel’s medium with 4% glucose was inoculated with primary culture at 5%, and the cultures were incubated at 28 °C for 24 h. Mycelia were collected by filtration through Mira cloth, and the collected mycelia were dried for 72 h at 70 °C and weighed.

For cellulase induction and production, RCM or Mandel’s medium with 2.5 g/L CaCO_3_ was used unless specified otherwise, and the experiments describing no calcium had no known source of Ca^2+^ added to RCM or Mandel’s medium. For transcriptomics and proteomics studies, strains were grown in Mandel’s medium for 48 h, and mycelia were collected at logarithmic phase and processed accordingly.

### Enzyme assays and biomass hydrolysis

Secretomes were evaluated for enzyme activities on 0.5% crystalline cellulose (Avicel, Sigma), 1% carboxymethyl cellulose (CMC, Sigma), 1% xylan (Himedia), and 1% starch (Himedia) as described previously [18]. Citrate–phosphate buffer (50 mM, pH 4.0) was used for diluting the secretomes and carrying out enzyme assays. Enzymes and substrates are incubated together for 30 min (2 h for crystalline cellulose) at 50 °C. The sugars released by enzyme action are measured by the dinitrosalicylic acid (DNSA) method. The absorbance at 540 nm was measured relative to a glucose or xylose standard curve. One unit of enzyme activity was defined as the amount of enzyme releasing 1 μmol of reducing sugar per min. *p*-nitrophenyl-β-d-glucopyranoside (pNPG) (Sigma) was used as a substrate to determine β-glucosidase activity in the secretome, and the amount releasing 1 μmol of *p*-nitrophenol per min was considered one pNPGase unit. The absorbance of the test at 410 nm was measured relative to the *p*-nitrophenol standard curve. Additionally, β-glucosidase activity was also detected by zymogram using 4-methylumbelliferyl-beta-D-glucuronide hydrate (4-MUG) (Sigma) as the substrate. Secretomes are electrophoresed on 10% Native PAGE; electrophoresed gels are rinsed with water and citrate–phosphate buffer (50 mM, pH 4.0), followed by incubation in 5 µM 4-MUG solution for one hour. The zymogram gels are visualized under UV light to detect fluorescence due to the release of 4-methylumbelliferone (4-MU). Then, the total cellulase activity of the secretome was measured by a filter paper assay that measures the fixed degree of conversion of substrate as previously described [18]. One filter paper unit is the amount of secretome that releases 2 mg glucose from 50 mg filter paper in 60 min at 50 °C.

Based on the protocol discussed in Ogunyewo et al. [19], secretomes were evaluated on nitric acid-pretreated rice straw using a 20% dry biomass substrate loading concentration at enzyme concentrations of 30 g/kg dry biomass weight (DBW) (3% enzyme load). The hydrolysis reaction was conducted in 1.2-ml 96-well plates with pretreated biomass at 20% DBW loading in a 250 μl final reaction volume. The protein content of the secretomes was measured, and an appropriate volume of 30 g/kg DBW was added to the reaction mixture. The reaction was conducted in 50 mM citrate–phosphate buffer (pH 4.0) and incubated at 50 °C with constant shaking at 200 rpm for 72 h. Control experiments were carried out under the same conditions using substrates without enzymes (enzyme blank) and enzymes without substrates (substrate blank); a substrate-free negative control was set up by filling wells with 50 mM citrate–phosphate buffer, pH 4.0, and the background of soluble sugars present in the respective biomass was determined by incubating each biomass in the absence of enzymes. Following the completion of hydrolysis, the plates were centrifuged at 3000 × g for 10 min in a swinging bucket centrifuge (Eppendorf, Germany) to separate the solid residue from the digested biomass. Hydrolysates were analyzed for their sugar content by high-performance liquid chromatography equipped with an Aminex HPX-87H anion exchange column (Bio-Rad, USA) and a refractive index detector, and the percentage release was calculated citing the theoretical yield.

### Transcriptomic studies by RNA-seq

NCIM1228 and ∆Mig1 were grown in Mandel’s medium with 4% glucose/Avicel. Total RNA was isolated from log phase cultures by a Qiagen Plant Mini RNeasy kit according to the manufacturer’s instructions. Traces of DNA, if any, are removed by DNase treatment before proceeding with RNA sequencing. RNA-seq was carried out on a HiSeq 2000 platform with 125 × 2 paired-end read chemistry (Bionivid Technology Pvt Ltd). Biological replicate sequencing libraries for both strains were created with poly-A tailed mRNA enrichment using the standard Illumina TruSeq mRNA RNA-Seq protocol. The RNA-Seq reads obtained were assembled using Trinity with a reference genome-guided approach. Assembled transcripts are quantified by mapping generated sequencing reads to the assembled transcripts using the alignment mapping program Bowtie2, and alignments are coordinate-sorted by SAMtools. The quantitative program RSEM generates fragments per kilobase of transcript per million mapped reads (FPKM) from the quantitated data. The protein domains were predicted using InterProScan. For differential expression profiling, all FPKM values were normalized to the library size using the R package Edge R. The obtained p-values were plotted against log_2_ fold change using Volcano R, and volcano plots were obtained to assess the significance of up- and downregulation of transcripts, as shown in the respective figures. The DNA binding and calcium-activated proteins were filtered and identified from the InterPro results. Heatmaps were generated from log_2_ FPKM values of selected transcription factors and calcium-activated proteins by performing hierarchical clustering using the Euclidean distance matrix option of the Morpheus heatmap tool.

### Expression profiling by real-time qPCR

For real-time PCR experiments, mycelia were harvested from log phase cultures, and total RNA was extracted using an RNeasy kit (Qiagen). Traces of DNA were removed by DNase (Invitrogen) treatment prior to cDNA synthesis, and the RNA concentration was measured by a NanoDrop. Equal amounts of RNA were used to synthesize cDNA with an Invitrogen cDNA synthesis kit. cDNA was used as a template to carry out qRT‒PCR using iTaq^™^ Universal SYBR^®^ Green Supermix (Bio-Rad) and a Bio-Rad CFX96 qPCR detection system. qRT‒PCR was performed in biological triplicates with tubulin as the endogenous control. Relative expression levels are normalized to tubulin, and fold changes in RNA level are the ratios of the relative expression level of test strain to control strain under repressing conditions and cellulase inducing conditions to carbon repressing conditions.

### Construction of gene overexpression and deletion cassettes

*Ctf1a* expression cassette: The construction of *ctf1a* expression cassette was reported in our previous study [7]. Briefly, *ctf1a* expression cassette was prepared by NEBuilder HiFi DNA assembly kit (NEB #E2621). A2553 bp region having promoter and ORF region of *ctf1a* (contig id 160_0.0) was assembled with 500 bp DNA fragments corresponding to upstream and downstream to *pyr4* ORF and the resultant cassette was cloned in pCambia1302, as mentioned earlier, at *Mau*BI and *Xho*I restriction sites [7]. The final binary vector of 10.7 kb size was transformed into *P. funiculosum* NCIM1228 by Agrobacterium-mediated transformation. For optimal growth of *ctf1a* transformants, growth media were supplemented with uracil.

*ctf1b expression* cassette: A 3953 bp DNA fragment containing the *ctf1b* gene along with a 1000 bp promoter and 200 bp terminator was PCR amplified from genomic DNA using primers Ctf1b Pro EcoRI F and Ctf1b Ter MluI R and cloned and inserted into pBIF, replacing T-DNA at *Eco*RI and *Mlu*I restriction sites. As a result, a 12.976 kb binary vector was created with *ctf1b* expression between the T-DNA arms.

*clr-2 overexpression cassettes*: The *clr-2* cassette (1005 bp promoter + 2753 bp *clr-2* gene + 207 bp terminator) was amplified from genomic DNA of NCIM1228 with Clr2PF1 and Clr2R2 primers. The fragment was cloned in pBIF at *Mun*I/*Bam*HI restriction sites, replacing the *gpd* promoter and *gfp* gene with the *clr-2* cassette in the T-DNA of pBIF. For *clr-2* under the Gpd promoter, 2753 bp *clr-2* gene + 207 bp terminator was amplified from genomic DNA of NCIM1228 with Clr2F1 and Clr2R2 primers and cloned at *Sac*I/*Bam*HI restriction sites. For *clr-2* expression under the *cbh1* promoter, the *cbh1* promoter was amplified from the NCIM1228 genome using Cbh1Pro-F and Cbh1 Pro-R and cloned and inserted into pBIF at *EcoRI/SacI* restriction sites replacing the *gpd* promoter, followed by *clr-2* cloning at *SacI/BamHI* restriction sites.

*ssp1 overexpression cassette*: First, the 1026 bp hygromycin resistance gene (*hph*) was replaced with the 573 bp nourseothricin resistance gene (*nat*) at the *Aat*II/*Eco*RI restriction sites in pCambia1302, resulting in pNat. The *ssp1* gene flanked by the glucoamylase promoter and terminator was chemically synthesized, and the resultant cassette of 5317 bp was cloned and inserted into pNat at *Eco*RI/*Mau*BI restriction sites.

### Western blot analysis

Cbh1 levels in the secretome were detected by western blotting as previously described [6]. For detection of intracellular Cbh1, mycelial extracts were collected by filtration using a mira cloth and washed three times with excess distilled water, and extra moisture was removed by pressing between the layers of filter paper before lyophilization. One hundred milligrams of finely ground lyophilized mycelia was added to 1 ml of lysis buffer (50 mM Tris–Cl, pH 8.0, 0.05% SDS, 0.1% sodium deoxycholate, 0.1% Triton X-100, 5 mM sodium pyrophosphate, 50 mM sodium fluoride, 0.1 mM sodium vanadate, 0.05% PMSF, cOmplete™ Mini, EDTA-free Protease Inhibitor Cocktail (Roche), and phosSTOP (Roche)) in bashing bead lysis tubes (Zymo Research), and the cells were lysed by 30 lysis cycles (1 min bead beater and 1 min ice) using cell disrupter (Genie). Mycelial extract was collected by centrifugation at 13000 rpm for 30 min. The whole mycelial extract was evaluated for its protein content by the BCA method. Fifty micrograms of whole mycelial protein were electrophoresed on 12% SDS‒PAGE gels, and the electrophoresed proteins were transferred to nitrocellulose membranes using a semidry blot technique. For western blotting, the membranes were blocked with 5% BSA in Tris buffer saline with 0.1% Tween-20 (TBS-T) for 2 h prior to incubation with rabbit anti-CBH1 (1:5000) overnight with regular shaking. The blots were washed with TBS-T three times and incubated with HRP-conjugated anti-rabbit/mouse for one hour. The blots were developed by G-biosciences ECL reagent, and chemiluminescence was detected using high-resolution chemiluminescence mode on a Bio-Rad ChemiDoc XRS + system.

### Supplementary Information

**Additional file 1.** FPKM values of shortlisted transcription factors in *P.* *funiculosum* NCIM1228 and ΔMig1 when cultured in glucose and cellulose.

**Additional file 2: Figure S1.** Over-expression of *ctf1a* in *P. funiculosum* NCIM1228. **Figure S2.** Over-expression of *ctf1b* and *clr-2* in *P. funiculosum* NCIM1228. **Figure S3.** Total cellulase activity of over-expression transformants. **Figure S4.** Ctf1a and Ctf1b are repressors of cutinase under cellulosic conditions. **Figure S5.** Clr-2 becomes non-functional without its fungal TF_MHR domain. **Figure S6.** Total cellulase activity of *ssp1* over-expression transformants. **Table S1.** List of strains used in the study. **Table S2.** List of plasmids used in the study. **Table S3.** List of primers used in the study.

## Data Availability

The RNA-seq dataset having FPKM values of shortlisted transcription factors from NCIM1228 and ∆Mig1 strains grown in glucose and Avicel are available in the Additional file 1.

## Abbreviations

2G: Second generation
TF: Transcription factor
FPU: Filter paper units
CaMKK: Calcium/calmodulin dependent kinase kinase
AMPK: AMP activated protein kinase
