# Supplementary material for: Calcium signaling positively regulates cellulase translation and secretion in a Clr-2-overexpressing, catabolically derepressed strain of Penicillium funiculosum
Source: Biotechnol Biofuels Bioprod. 2024 Feb 9;17:21. doi: 10.1186/s13068-023-02448-3 (PMC10858516; doi:10.1186/s13068-023-02448-3)
Supplement: Supplementary file 2 — Additional file 2: Figure S1. Over-expression of ctf1a in P. funiculosum NCIM1228. Figure S2. Over-expression of ctf1b and clr-2 in P. funiculosum NCIM1228. Figure S3. Total cellulase activity of over-expression transformants. Figure S4. Ctf1a and Ctf1b are repressors of cutinase under cellulosic conditions. Figure S5. Clr-2 becomes non-functional without its fungal TF_MHR domain. Figure S6. Total cellulase activity of ssp1 over-expression transformants. Table S1. List of strains used in the study. Table S2. List of plasmids used in the study. Table S3. List of primers used in the study. [file 13068_2023_2448_MOESM2_ESM.docx]

Additional Information for

Ca^2+^ signaling positively regulates cellulase translation and secretion in Clr-2-overexpressing, catabolically derepressed strain of *Penicillium funiculosum*

Additional Fig. S1 Over-expression of *ctf1a* in *P. funiculosum* NCIM1228.

Additional Fig. S2 Over-expression of *ctf1b* and *clr-2* in *P. funiculosum* NCIM1228.

Additional Fig. S3 Total cellulase activity of over-expression transformants.

Additional Fig. S4 Ctf1a and Ctf1b are repressors of cutinase under cellulosic conditions.

Additional Fig. S5 Clr-2 becomes non-functional without its fungal TF_MHR domain.

Additional Fig. S6 Total cellulase activity of *ssp1* over-expression transformants.

Additional Table S1. List of strains used in the study

Additional Table S2. List of plasmids used in the study

Additional Table S3. List of primers used in the study.


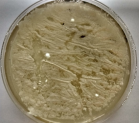

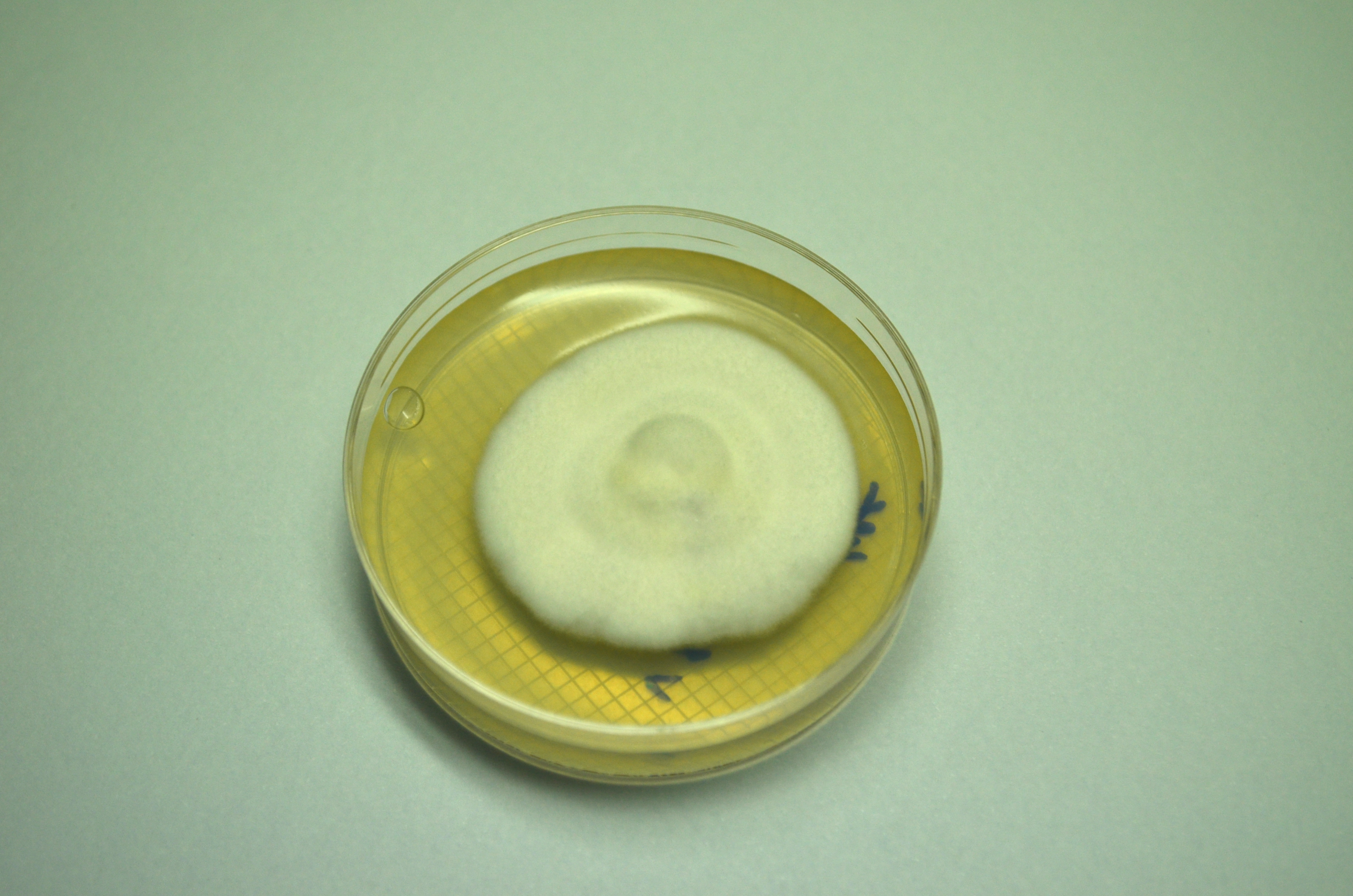

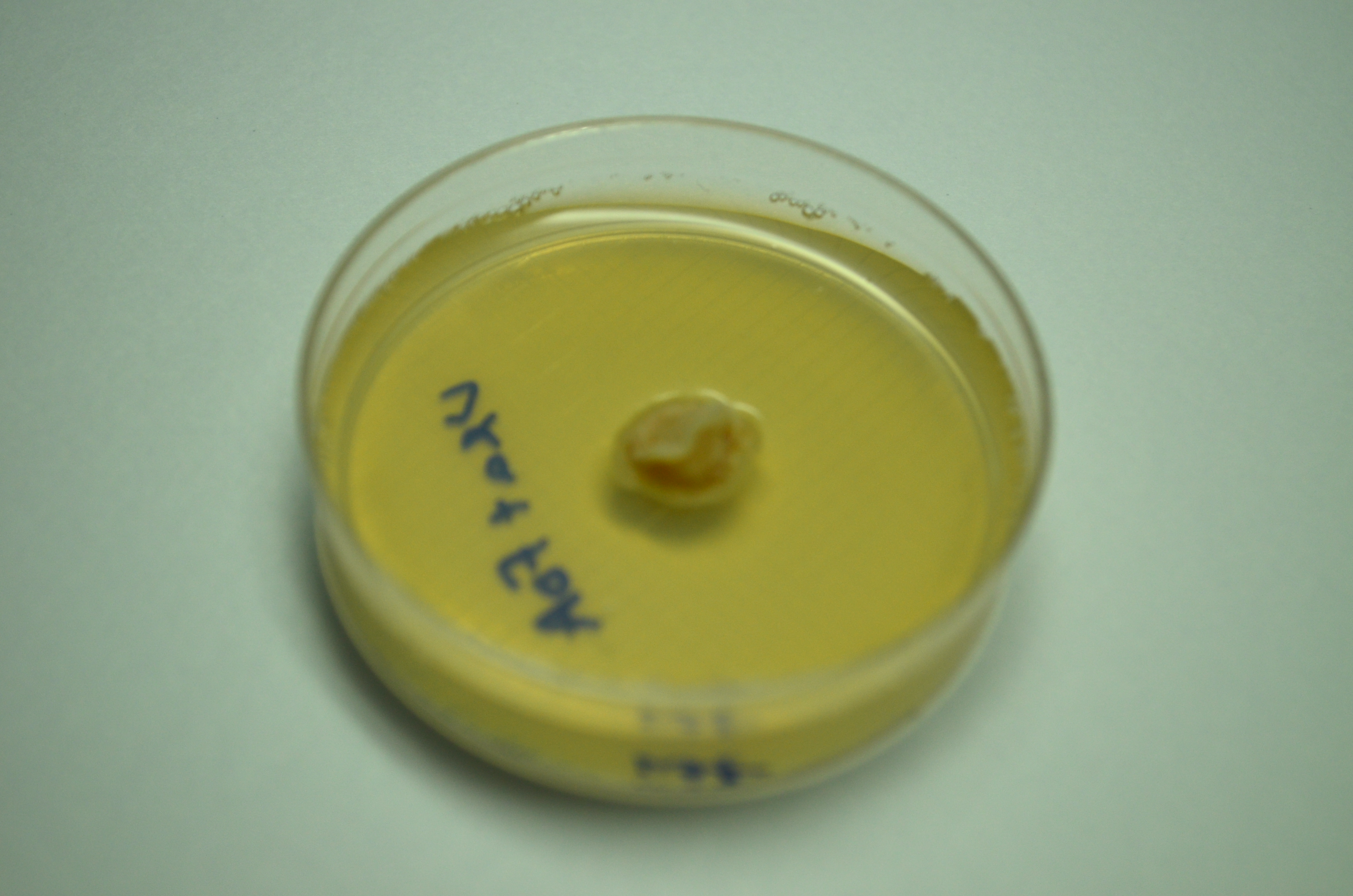


NCIM1228

Ctf1a/NCIM1228

SC Ura + 5-FOA


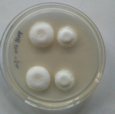

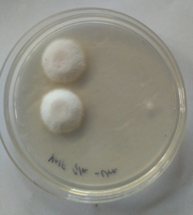


SC + Ura

SC - Ura

NCIM1228

Ctf1a/NCIM1228


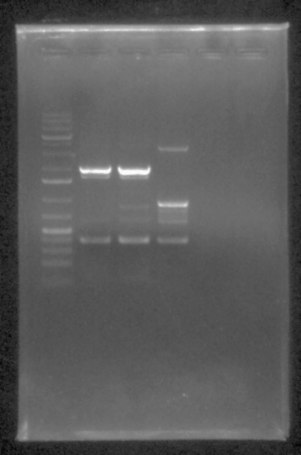


*pyr4* (2071 bp)

*ctf1a expression cassette* (3471 bp)

2 kb

4 kb

DNA ladder

C

T1

a

b

d

c

e


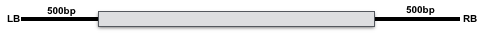


*ctf1* pro + ORF (3086 bp)

*pyrG* upstream homology region

*pyrG* downstream homology region

*ctf1a* expression cassette (4090 bp)

Xba1 pyrG-up F

pyrG-up R

Ctf1a F

Ctf1a R

pyrG-dn - F

MauB1 pyrG-dn - R

**Additional Fig. S1 Over-expression of *ctf1a* in *P. funiculosum* NCIM1228.** a, *ctf1a* expression cassette and the primers pairs used to construct the cassette by NEB builder assembly. *ctf1a* expression cassette was used to replace 1686 bp ORF region of *pyrG* gene. b, SC+Ura+5-FOA plate having positive transformants. c, growth of *ctf1a* transformants in presence of 5-FOA (2 mg/ml). d, growth phenotype of NCIM1228, and ctf1a transformants in absence of uracil. e, PCR confirmation of *ctf1a* transformants using pyrG 180 up F and PyrG 205dn R primers, Control (C) taken is NCIM1228 having native copy of pyrG with amplicon size of 2071 bp, and the ctf1a transformants have amplicon size of 3471 bp.


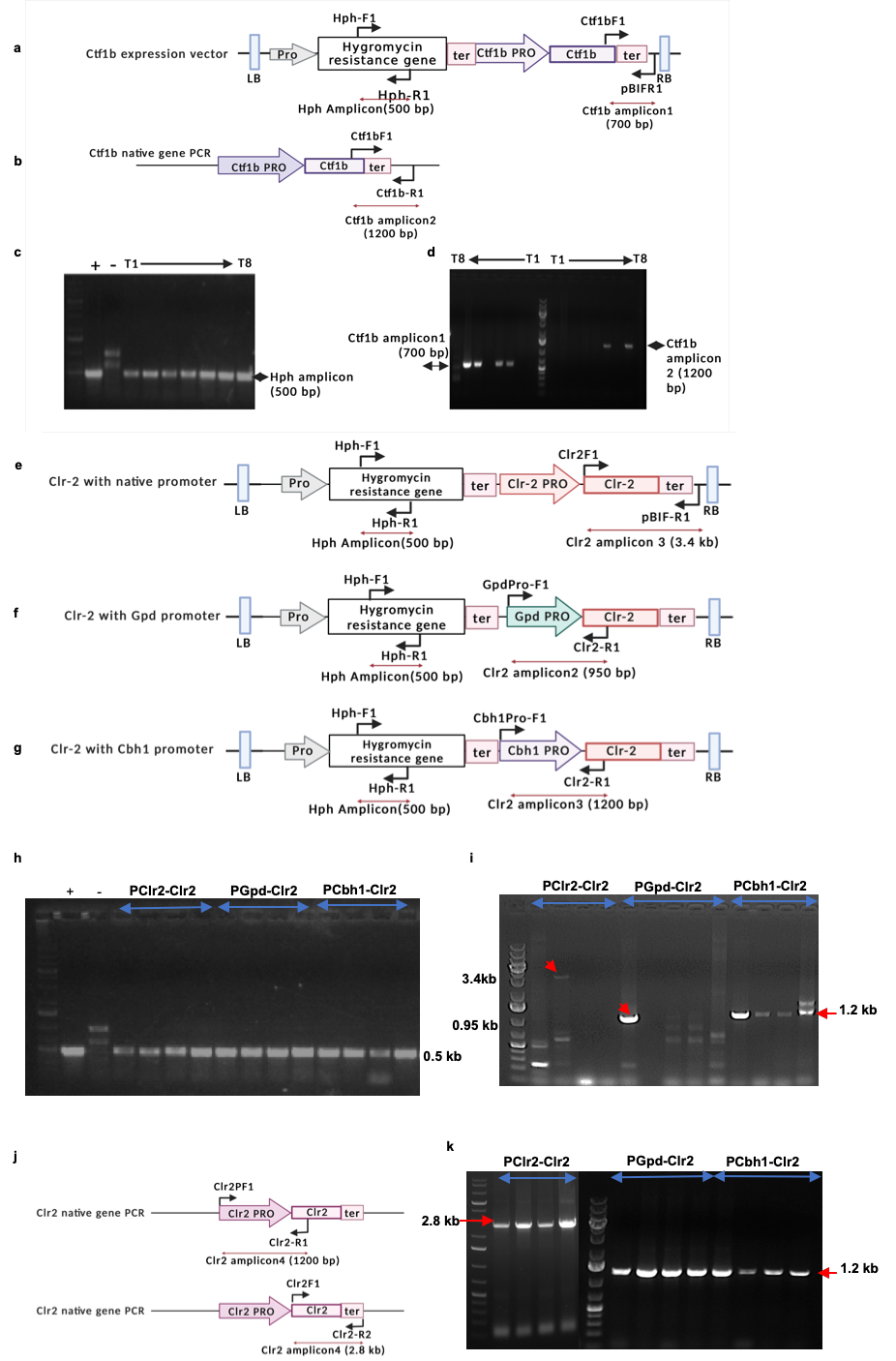


Additional Fig. S2 Over-expression of *ctf1b* and *clr-2* in *P. funiculosum* NCIM1228. a, *ctf1b* expression cassette and the primers pairs used to confirm the cassette integration in the genome. b, Primers used to amplify the native copy of *ctf1b*. c, PCR confirmation of *ctf1b* transformants using *hph* primers, and (d) presence of at least two copies of *ctf1b* in the genome (native and transformed). *clr-2* expression cassettes with (e) *clr-2* native promoter, (f) *gpd* promoter, and (g) *cbh1* promoter. h, PCR confirmation of *clr-2* transformants using *hph* primers, and (i) presence of a transformed copy of *clr-2*. j, Primers used to amplify the native *clr-2* gene, and (k) PCR confirmation of native *clr-2*, apart from the transformed *clr-2*.


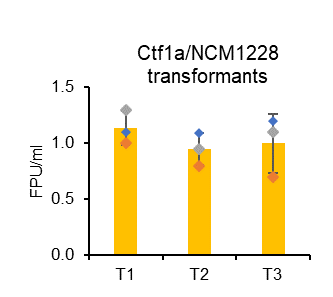

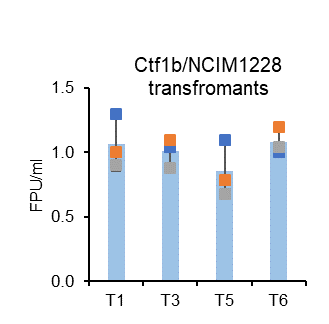

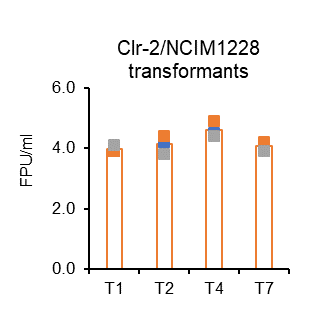

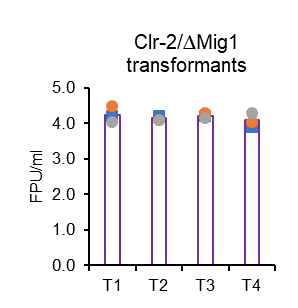


a

b

c

d

Additional Fig. S3 Total cellulase activity exhibited by over-expression transformants of (a) *ctf1a*, (b) *clr-2,* (c) *ctf1b* in *P. funiculosum* NCIM1228. (d) Total cellulase activity exhibited by over-expression transformants of *clr-2* in ∆Mig1.


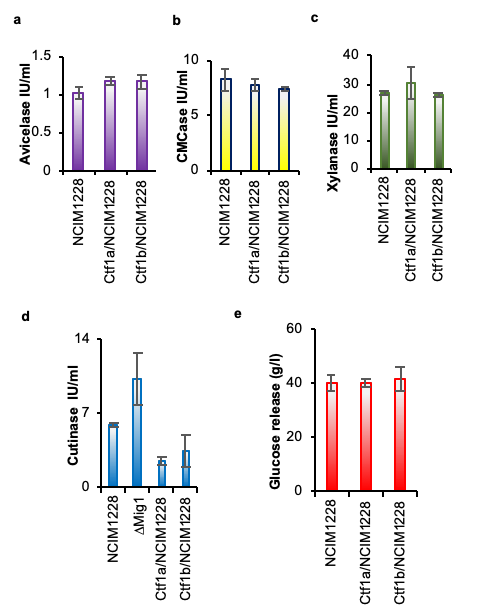


Additional Fig. S4 Ctf1a and Ctf1b are repressors of cutinase under cellulosic conditions. Two transformants each of Ctf1a/NCIM1228, and Ctf1b/NCIM1228 along with NCIM1228 (control) were grown in cellulosic growth medium for 6 days and secretome collected by centrifugation was checked for activities of (a) Exocellulase (Avicelase), (b) Endocellulase (CMCase), and (c) Xylanases, and (d) cutinase (∆Mig1 used as additional positive control). (e) Biomass hydrolyzing capacity was determined by the release of glucose from 10% (dry biomass weight) acid pre-treated sugarcane bagasse incubated with 30mg/ml secretome protein for 3 days. All the experiments were performed in triplicates.


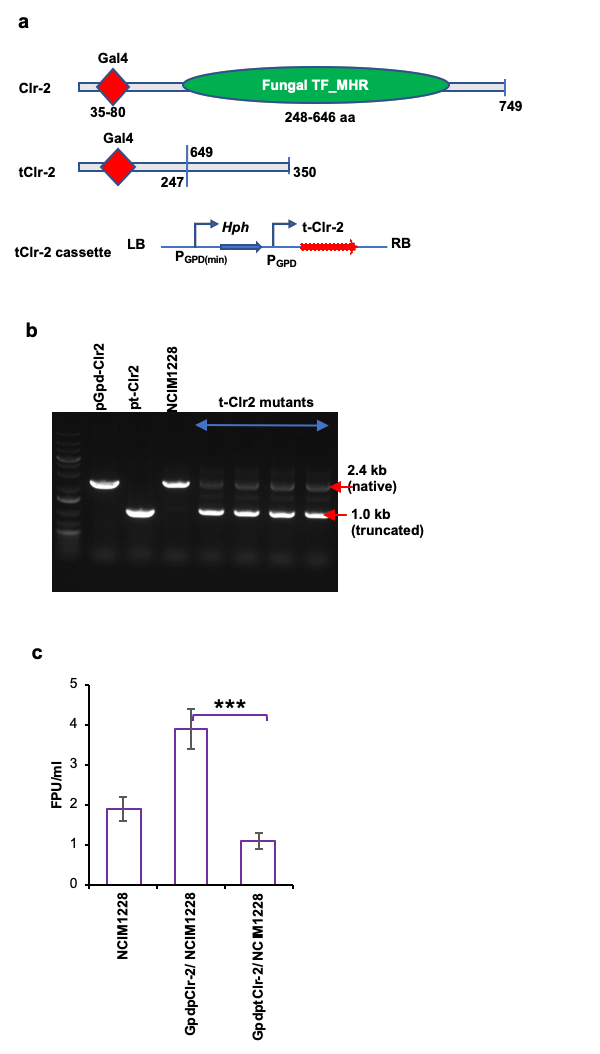


Additional Fig. S5 Clr-2 becomes non-functional without its fungal TF_MHR domain. (a) Schematic representation of domain structure of Clr-2 protein (upper panel), the truncated Clr-2mutant protein (tClr-2) and T-DNA having *tclr-2* under a strong constitutive *gpd* promoter (lower panel). (b) PCR with Sac1 Clr-2F and BamH1 Clr-2R confirmed amplification of *tclr-2* gene in addition to native *clr-2* in the genome of transformants. (c) Secretomes of NCIM1228, Clr-2/NCIM1228 and tClr-2/ NCIM1228 were tested for total cellulase activity when grown in the cellulosic medium for 5 days.


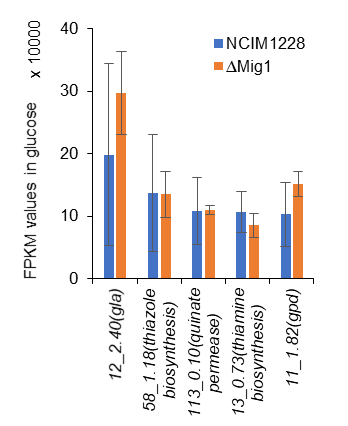

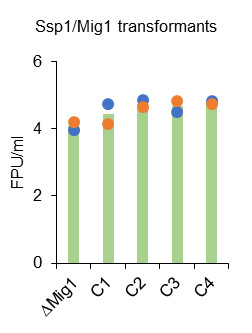

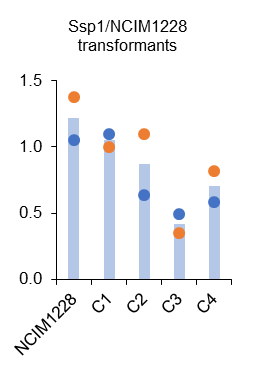


a

b

c

Additional Fig. S6 *ssp1* over-expression in *P. funiculosum*. (a) Transcript levels of five most expressed genes in *P. funiculosum* NCIM1228 and ∆Mig1 cultured in glucose. Total cellulase activity measured by filter paper unit assay of (b) Ssp1/NCIM1228 , and (c) Ssp1/ ∆Mig1 transformants.

Additional Table S1. List of strains used in the study

| S. No | Species | Strain/Genotype |
| --- | --- | --- |
| 1 | *E. coli* | DH5α |
| 2 | *Agrobacterium tumefaciens* | LBA4404 |
| 3 | *Penicillium funiculosum*  Ctf1a/NCIM1228 (∆PyrG)  Ctf1b/NCIM1228  P_clr2_Clr-2/NCIM1228  ∆Mig1 (PfMig1^88^)  _Pclr2_Clr-2/∆Mig1  _Pgpd_Clr-2/∆Mig1  _Pcbh1_Clr-2/∆Mig1  _Pgla_Ssp1/NCIM1228  _Pgla_Ssp1/∆Mig1  _Pcbh1_Clr-2/P_Gla_Ssp1/∆Mig1 | NCIM1228  *pyrG*::*ctf1a* (Randhawa et al, 2021)  *hph*,*ctf1b* (This study)  *hph*,*clr-2* (This study)  *mig1*::*ble* (Randhawa et al, 2018)  *mig1*::*ble, hph*,_PClr2_*Clr-2* (This study)  *mig1*::*ble, hph*,_PGpd_*Clr-2* (This study)  *mig1*::*ble, hph*,_PCbh1_*Clr-2* (This study)  *Nat*,P_Gla_*ssp1* (This study)  *mig1*::*ble,* *nat*,_PGla_*ssp1* (This study)  *mig1*::*ble,* *nat*,_PGla_*ssp1*, *hph*,_PCbh1_*Clr-2* (This study) |

Additional Table S2. List of plasmids used in the study

| S. No | Plasmids | Genotype |
| --- | --- | --- |
| 1 | Gene overexpression  pCTf1a  pP_ctf1b_Ctf1b  pP_Clr2_Clr-2  pP_Gpd_Clr-2  pP_Cbh1_Clr-2  pP_Gla_Ssp1 | pCambia1302 _PGpd_*hph,*_PGpd_*gfp*::_PpyrG,Pctf1a_*ctf1a*-t_PyrG_  pBIF _Pgpd_*gfp*::_Pctf1b_*ctf1b*  pBIF _Pgpd_*gfp*::_Pclr2_*clr-2*  pBIF *gfp*::*clr-2*  pBIF _PGpd_*gfp*::_PCbh1_*clr-2*  pBIF P_Gpd_*hph::*P_Gpd_*Nat,* P_Gpd_GFP::P_Gla_Ssp1-t_Gla_ |

Additional Table S3. List of primers used in the gene over-expression

| S. No | Primer Name | Sequence |
| --- | --- | --- |
| 1 | pBIF primers  Hph-F1  Hph-R1  GpdPro-F1  pBIFR1 | AGATCGTTATGTTTATCGGCAC  GCTGTTATGCGGCCATTGTC  GAATTCTGTACAGTGACCGGTGACTC  GCCAAGCTTCCTCTAAACAAGTGTACCTGTGC |
| 2 | *ctf1a gene*  Xba1 pyrG-500up F  pyrG-up-R  Ctf1a F  Ctf1a R  pyrG- dn- F  MauB1 pyrG-500dn-R  PyrG-180upF  PyrG205dn R | acgcTCTAGACGCCCACGGCAATCTAGTCG  GTGCAAGGCACTCTGTAGATCGTGAATATTAGTTGGTCTTTAAAG  CACGATCTACAGAGTGCCTTGCACTAATTGCGTG  GATGATAAAAGATAAACTCATGAGCTCGCCAGCCATGGGGTCGGC  GCCGACCCCATGGCTGGCGAGCTCATGAGTTTATCTTTTATCATC  acgcCGCGCGCGTACAAAGTGCTATCCCTATCAATGAAATTACCAG  GGTTTGAGACTGGCATCTTGAATG  GCGTTGCAGGAGTTGACTTG |
| 3 | *ctf1b* gene  Ctf1bF1  Ctf1bR1  Ctf1b 200 R | GCCGGAGAAAAAAATTTCGTGTCG  ACGTACGCGTTCATAATTTCAGGGATAGACCCATGTCAG  AATTCAATGGATTCTTGGAATGGCG |
| 4 | *clr-2* gene  Clr2PF1  Clr2F1  Clr2-R1  Clr2-R2  Cbh1Pro-F1  Cbh1 Pro-R | ACGTCAATTGTCTCCGCGAGCGGATGG  AGACGAGCTCATGTTTCTCACATTCGAGTCCTC  TCAGCCTTCTTGTACCACCG  ACGTGGATCCTGTCTTGTTTTTGAGGAGAGGGATGGC  ATGTGAATTCTTTCATTTTAATTTGTCAGACAGC  ACGTGAGCTCTGTGTCGATTGCTTCTGACTGTTG |
| 5 | *ssp1* gene  Amylase Pro F  Amylase Ter R  Ssp1 F  Ssp1 R | CTCCACTTATTAGAGCCAGATATAGCTTCAC  TCCCTCGGAAAATAACAACATAAGCAGTAG  CTCGATCGTCCCTAACCAATCCTG  CAGACCTGGAGTAGAAATCGGGG |
| 6 | qPCR Primers  CBHI F  CBHI R  CBHII F  CBHII R  EG-GH5 F  EG-GH5 R  EndoGH45 F  Endo GH45 R  Xyl1 F  Xyl1 R  Xyl2 F  Xyl2 R  Xyl3 F  Xyl3 R  Bgl1 F  Bgl1 R  Bgl2 F  Bgl2 R  Actin F  Actin R  Tubulin F  Tubulin R  Ssp1 mRNA F  Ssp1 mRNA R  Ctf1b mRNA F  Ctf1b mRNA R  Clr2 mRNA F  Clr2-R1  Ctf1a mRNA F  Ctf1a mRNA R  Intron (-ve control)  intron F  intron R | GCAAACACGAAGCTGGTATGG  GGTGACATAGGAGCTGCCGG  CTGGGGACAATGTGGCGG    GTTGTCGGCGGCGATGTC  GCAACCATTGGTGAATTCATCAGTCAG    CTGCCATTGTACCTTCCATAATTGTGAG  GATTCCTGGCAGCTCGGC    AGGTTGGTGACCATAACGATGATG  GTGCTGCTCGCTCCATTACCTAC  CTTGGTAGGTGCCACCGTCAG  GCAATGAAATGGCAACCCACCG  CCTTCAAGGCAGCAATAAGGGTTG  GGTGATGCTAACCCCATCACCTAC  AGATATCATAGGTGCCGCCGTC  CTGGCGAAGGGTACATAACAGTCG  GCCAGCCCATACTACGGC  CAATGACACTGTTAGATATGCCATCTCAG  GACCACGCGGCTTGACC  CATTGTCATGTCTGGCGGTACTAC  CGTACTCCTGCTTGGAGACCC  ATTGCTCAGGTTGTCTCCTCCATC  ATTGCTCAGGTTGTCTCCTCCATC  CTCGATCGTCCCTAACCAATCCTG  CAGACCTGGAGTAGAAATCGGGG  GCCGGAGAAAAAAATTTCGTGTCG    AATTCAATGGATTCTTGGAATGGCG  ATGTTTCTCACATTCGAGTCCTC  TCAGCCTTCTTGTACCACCG  ATGCCGGCGAACACATTG  GGATAATGAACGACATCTGTGAAGCC  GTCAGTGGCCAAACATCACTACTG  GGATGACAATCCATGGAGGTCGTATG |
